# Diagnosis and surgical approach of congenital Gerbode defect: A case report

**DOI:** 10.1016/j.ijscr.2024.109718

**Published:** 2024-04-27

**Authors:** Paula L. Torres Gómez, Federico Núñez, Giovanny Ríos, Isabella Van-Londoño

**Affiliations:** a Cardiovascular Surgery Department, Fundación Clínica Shaio, Bogotá, Colombia; b Faculty of Medicine, Pontificia Universidad Javeriana, Bogotá, Colombia; c School of Medicine and Health Sciences, Universidad del Rosario, Bogotá, Colombia

**Keywords:** Cardiac septal defects, Ventricular cardiac defects, Interventricular communication, Cardiovascular surgery, Gerbode defect

## Abstract

**Introduction:**

The Gerbode defect is an infrequent cardiac anomaly, with an incidence below 1 % in current worldwide literature. It consists of a communication between the left ventricle and right atrium in the membranous septum with consequential hemodynamical and structural heart changes and can present as either congenital or acquired. The concomitant affectation of the tricuspid valve poses its difficulty as a diagnostic and therapeutical challenge.

**Presentation of case:**

This case report presents a 27-year-old patient with an incidental finding of congenital Gerbode defect with hemodynamical repercussion during surgical treatment for multidisciplinary management in the context of open thoracic trauma.

**Discussion:**

This defect has been infrequently described in the literature, and although several classification methods have been proposed, its diagnosis is challenging and must be considered when faced with nonspecific cardiac systems.

**Conclusion:**

It reports an infrequent congenital heart defect posing as traumatic or postoperative, generating a challenging diagnosis and successful surgical treatment given to the patient using a multidisciplinary approach to further broaden scientific literature on such an underrepresented pathology.

## Highlights

• Gerbode defect is a communication between the left ventricle and right atrium.
• It occurs in less than 1 % of the population, 7 % of them being congenital.
• We present the case of a man with a congenital defect admitted for thoracic trauma.
• The patient was taken to successful defect correction through right atriotomy.

## 1 Introduction

The Gerbode defect/communication is a very infrequent pathology (with an incidence below 1.0 % reported worldwide) which courses as mainly asymptomatic and is of late progression. As such, initial management strategies to reach to its diagnosis must include an integral multidisciplinary evaluation and high clinical suspicion when faced with structural cardiac changes such as biatrial enlargement, so the patient can be taken to a possible posterior surgical approach with adequate outcomes [[1], [2], [3]]. Here we present the case of a 27-year-old patient with an incidental finding of congenital Gerbode defect with hemodynamical repercussion during surgical treatment for multidisciplinary management in the context of open thoracic trauma.

## 2 Presentation of case

We present the case of a male patient of 27 years of age admitted to our institution due to an early postoperative remission for an appointment with the cardiovascular surgery group. Medical history from the remission center reported he was admitted due to hypovolemic shock secondary to open thoracic trauma, wounded by a sharp weapon in the precordial region. The patient required initial basic life support maneuvers and was taken to the operation room where a sternotomy requiring neurorrhaphy of the superior right lobe and cardiorraphy of the right atrium was performed, coursing with adequate postoperative evolution. However, during the initial follow-up appointment an echocardiogram was performed with findings of an aorto-atrial fistula with suspicion of a posttraumatic interventricular communication. Considering these findings, the patient was remitted to our institution.

The patient progressed with growing decline in functional class, presenting with a New York Heart Association (NYHA) class type III/V. He had a previous history of myocarditis of unknown origin untreated in 2010, and an intracardiac conduction block with indication for definitive artificial cardiac pacemaker implantation, which was previously dissented by the patient. Additionally, he had a history of daily cannabis consumption.

**Fig. 1:**
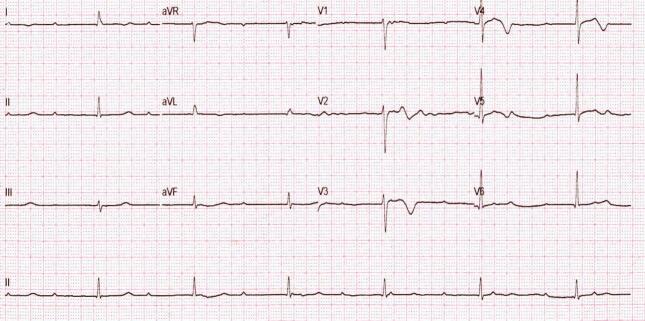
“Patient's electrocardiogram”. Evidence of a third-grade atrioventricular block.

During the patient's initial evaluation in the Cardiovascular Intensive Care Unit (CICU), bradycardic cardiac rhythms with a grade III/IV holosystolic murmur was auscultated, alongside an effacement of the first heart sound, reduced right vesicular murmur extending to the middle third of the right hemithorax and a third-grade atrioventricular block, evidenced in an electrocardiogram. The patient's electrocardiographic tracing can be seen in Fig. 1.

**Fig. 2:**
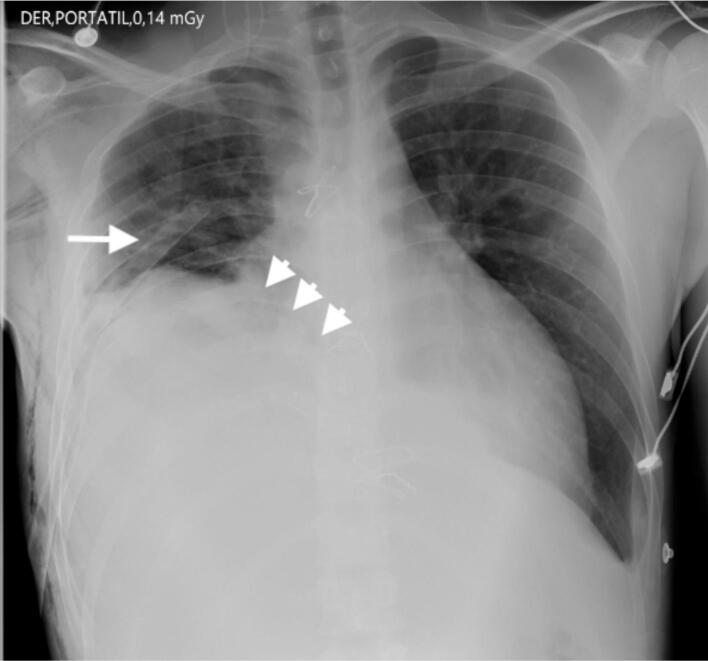
“Chest X-Ray (PA)”. Diffuse opacity in right lung base with lobar atelectasis and pleural effusion (arrowheads) with right diaphragm elevation secondary to a thoracostomy tube (arrow).

Patient was taken to a chest X-ray (Fig. 2), which evidences a poorly defined opacity in the basal right lung with signs of reduced volume secondary to lobar atelectasis and effacement of the ipsilateral costophrenic angle due to a pleural effusion of moderate amounts associated to an elevation of the right diaphragm due to a right thoracostomy tube.

**Fig. 3:**
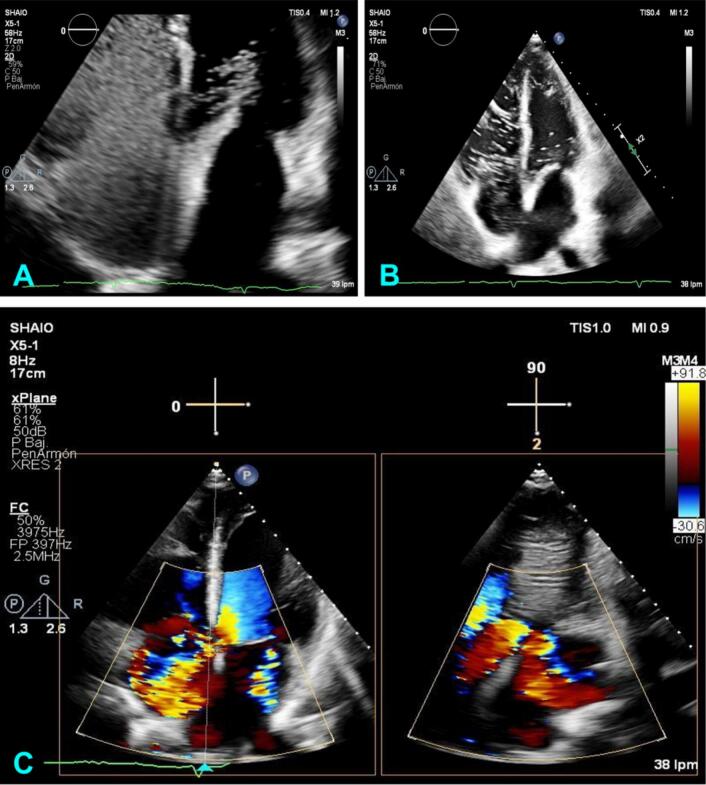
“Transthoracic echocardiogram”. A-B. Study contrasted using shaken saline solution with continuity solution of 7 mm with left ventricle to right atrium doppler flow, negative contrast in the right atrium and bubble movement to the left ventricle during diastole.

A transthoracic echocardiogram (Fig. 3) was taken, which reported the following: Biatrial enlargement (left atrium 44 m1/m2 – right atrium 35 m1/m2), left ventricle of usual size and morphology, without segmental contractility disorders and preserved systolic function (left ventricular ejection fraction [LVEF] 65 %). Enlargement of the right cardiac chambers with preserved right ventricle systolic function and signs of volume overload, massive functional tricuspid insufficiency (Calculation of the effective regurgitant orifice [ERO] 0.72 cm^2^, 70 ml regurgitating volume) with eccentric jet directed towards the right atrium's lateral wall. Study was complemented using shaken saline solution as contrast fluid, observing a Gerbode type defect.

**Fig. 4:**
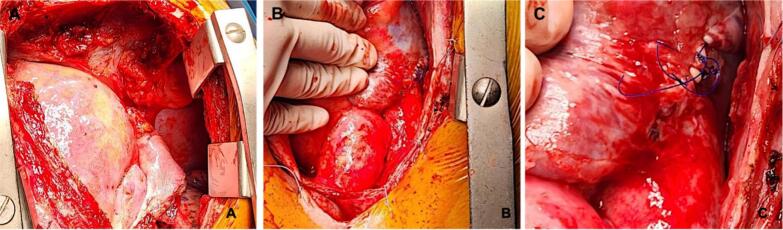
“Intraoperative photographs”. Chamber dilation due to excessive flow (A) and extra-institutional right atrial cardiorrhaphy (B, C).

**Fig. 5:**
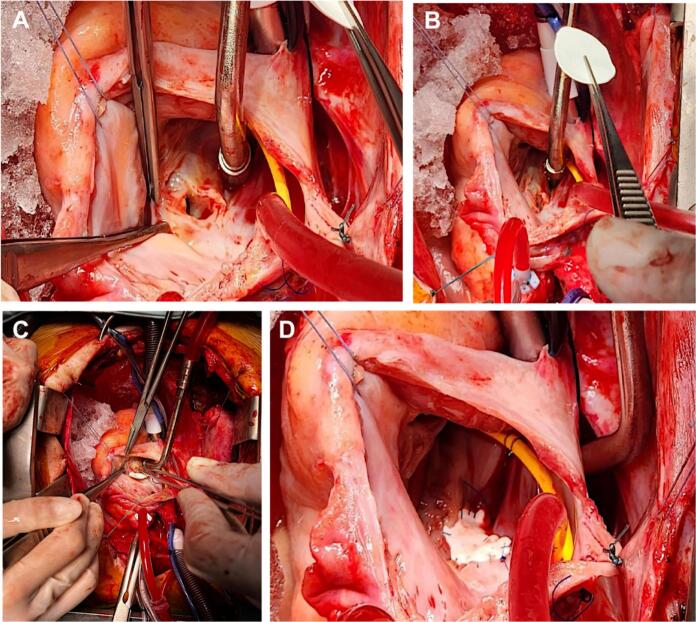
“Intraoperative photographs”. A defect of defined borders and a 7 mm diameter fibrosis communicating the left ventricle with the right atrium can be observed through right atriotomy (A-C). The PFTFE patch layout and defect closure can be seen (D).

Considering clinical and imaging findings, performing surgical closure of Gerbode's defect was decided. The patient was taken to a second median sternotomy, with conventional canulation using Extracorporeal Membrane Oxygenation (ECMO) and del Nido cardioplegic solution, maintaining normothermia. The following findings were identified: structural changes of the right chambers, previous extra-institutional atrial cardiorrhaphy (Fig. 4 [A, B, C]), tricuspid ring dilation and a defect communicating the left ventricle with the right atrium above the tricuspid valve (Fig. 5 [A-D]).

Surgical approach was through right atriotomy, partially detaching the tricuspid's septal valve, performing defect closure using a polytetrafluoroethylene (PTFE) patch, after which the septal valve was reinserted using 5/0 polypropylene sutures (Fig. 5-B), alongside conventional suturing of the atrium. ECMO times were of 75 min and aortic clamp was 53 min, respectively.

The patient was easily released from the bypass, a follow-up transesophageal echocardiogram reported satisfactory closure of the tricuspid valve and of Gerbode's defect. The patient was transferred to the intensive care unit where he coursed with an adequate evolution, devoid of complications. On the eighth postoperative day a definitive cardiac resynchronization therapy pacemaker with coronary sinus stimulation was implanted. Patient was given hospital discharge on postoperative day 10.

## 3 Discussion

The Gerbode's defect is an infrequent pathology consisting of an anomalous communication between the left ventricle and the right atrium. It was first described by Thurman J. in 1838 but was attributed to the professor Frank Gerbode after performing a presentation of 5 cases with successful surgical repair in 1958 [[4], [5], [6]]. It is of varied etiology and may be acquired by complication of endocarditis, stroke, and valve repair procedures or due to genetic anomalies [4,6]. The latter represent 0.08 % of etiologic rates and its pathogenesis is associated with sequencing alterations of the following genes: NK2 Homeobox 2 (NKX2-5), GATA4, and T-box 5 transcription factor (TBX5) [4,7]. Other etiologies are here reported according to their frequency: postsurgical defect 52 %, endocarditis 19 %, congenital 7 % and ischemic 3 % [6].

Clinical symptoms are unspecific, but dyspnea and edema secondary to right atrium overload are the most frequent [4], but can course as asymptomatic in 21 % of acquired defects and in 25 % of congenital defects [8,9]. During the physical exam it is possible to auscultate a grade III-VI holosystolic murmur, not modified by breathing in the third and fourth intercostal parasternal left spaces [6].

Diagnostic method of choice is the transesophageal echocardiogram with a high velocity systolic jet in the right atrium, while being careful it is not confused with tricuspid regurgitation [4,6,7,9]. Another important finding is enlargement of the right chambers. A cardiovascular magnetic resonance can be used to define the defect more precisely by measuring right and left chambers and jet flow from the left ventricle [3]. A cardiac computerized tomography angiogram is another plausible option for obtaining detailed anatomical knowledge of the defect [10].

Riemenschneider and Moss initially classified the defect into two functional types: direct when it goes through the membranous septum and indirect if there is a defect in the ventricular septum with consequential tricuspid regurgitation [4,11]. These terms were further modified according to the anatomical relationship of the anomaly to the tricuspid valve into supravalvular when occurring in the atrioventricular septum (occurring in one third of cases) and infravalvular (occurring in two thirds of cases). Sakakibara and Konno included a third type as intermediate when there were both supravalvular and infravalvular components [12]. The incidence between these three types is of 76 %, 16 % and 8 %, respectively according to Yuan [13].

Treatment depends on symptom severity, septal defect size, coexisting anomalies, flow rate and comorbidities [3,6,9]. In asymptomatic, chronic cases with a small defect, treatment can be conservative [3]. Traditionally, congenital or acquired defects require surgical treatment consisting of closing the abnormal communication using pericardium, Dacron or Amplatzer patches [3,4]. Some authors recommend surgical correction regardless of defect size to lower the risk of infectious endocarditis [3]. A right atriotomy approach allows for correction of the septal defect and tricuspid leaks with satisfactory results [11].

The case described is relevant considering the different analysis and interpretation uncertainties it provoked from its initial diagnostic approach and image interpretation. The patient's context, including his history of an atrioventricular block, cardiac trauma and recent surgical intervention constituted confusion variables for reaching the correct diagnosis. When analyzing these anomalies, we consider that a thorough study of echocardiographic images and multidisciplinary management given to the patient is crucial to evaluate the defect's characteristics and their clinical and hemodynamical repercussion. The documented intraoperative findings for right chamber enlargement and fibrotic changes in the defect's ostium (atrium) allowed to establish its temporality as a chronic anomality, which allowed for its classification into a congenital and not acquired anomaly, highlighting the academic impact of this case considering its difficult diagnosis and successful management provided.

Limitations for this case report include not having detailed information from the patient's remission center and the lack of any long-term follow-up data on the patient. This work was reported following the SCARE criteria and the guidelines checklist has been completed by the authors for this case report, attached as supplementary material [14].

## Consent

Written informed consent was obtained from the patient before doing this manuscript for both case description and obtained images, approving the publication of this manuscript. Information revealing the subject's identity was avoided.

## Ethical approval

Ethical compliance with the World Medical Association Declaration of Helsinki, current legislation on research Res. 008430-1993 and Res. 2378-2008 (Colombia) and the International Committee of Medical Journal Editors (ICMJE) were ensured under our Ethics and Research Institutional Committee (IRB) approval.

Ethical approval for this study (Ethical Committee N° CEI 031, Act 380) was provided by the Abood Shaio's Foundation Ethics in Research Committee, Bogotá, Colombia on March 13 2024.

## Funding

This research did not receive any specific grant from funding agencies in the public, commercial, or nonprofit sectors.

## Author contribution

Paula L. Torres Gómez: Study concept and design, data collection, data analysis, drafting of manuscript

Federico Núñez: Data collection, critical revision of manuscript

Giovanny Ríos: Critical revision of manuscript

Isabella Van-Londoño: Drafting of manuscript, critical revision of manuscript

## Guarantor

Paula L. Torres Gómez.

## Conflict of interest statement

The authors declare that they have no known competing financial interests or personal relationships that could have appeared to influence the work reported in this paper.

## Data Availability

All data generated or analyzed during this study are included in this article and its supplementary material. Further enquiries can be directed to the corresponding author.
